# Posterior Reversible Leukoencephalopathy Syndrome and Disseminated Varicella‐Zoster Virus Infection After Kidney Transplantation

**DOI:** 10.1002/iju5.70030

**Published:** 2025-06-04

**Authors:** Kenji Tsutsui, Shigeaki Nakazawa, Makoto Kinoshita, Yoko Higa, Soichi Matsumura, Shota Fukae, Ryo Tanaka, Norichika Ueda, Yoichi Kakuta, Norio Nonomura

**Affiliations:** ^1^ Department of Urology Osaka University Graduate School of Medicine Osaka Japan; ^2^ Department of Neurology Osaka University Graduate School of Medicine Osaka Japan

**Keywords:** disseminated varicella‐zoster virus infection, kidney transplantation, posterior reversible leukoencephalopathy syndrome

## Abstract

**Introduction:**

Posterior reversible leukoencephalopathy syndrome (PRES) is a rare but serious complication in kidney transplant recipients, often triggered by calcineurin inhibitors (CNIs) and infections.

**Case Presentation:**

A 52‐year‐old woman with end‐stage kidney disease underwent cadaveric renal transplantation. Two months post‐transplant, she presented with headaches, visual disturbances, hypertension, and altered consciousness. Cranial MRI confirmed PRES. After conversion from tacrolimus to cyclosporine and antihypertensive therapy, symptoms improved. However, the patient developed disseminated varicella‐zoster virus infection, resulting in meningitis. Treatment with acyclovir and reduction of immunosuppression led to full recovery without recurrence.

**Conclusion:**

This case highlights the importance of recognizing PRES and its triggers, including infections and CNIs, in kidney transplant recipients. Early diagnosis and appropriate management are crucial for preventing severe outcomes.

**Summary**

- A renal transplant recipient manifested posterior reversible leukoencephalopathy syndrome and disseminated varicella‐zoster virus after kidney transplantation.

**Abbreviations**
CNI: calcineurin inhibitor
CSF: cerebrospinal fluid
CT: computed tomography
DWI: diffusion‐weighted imaging
MMF: mycophenolate mofetil
MRI: magnetic resonance imaging
PRES: posterior reversible leukoencephalopathy syndrome
SOT: solid organ transplantation

## 1 Introduction

Posterior reversible leukoencephalopathy syndrome (PRES) is a neurologic disorder characterized by vasogenic edema, predominantly affecting the occipital and parietal lobes [1]. Common symptoms include altered consciousness, seizures, headaches, and visual disturbances. PRES is a rare but serious complication following kidney transplantation, often associated with CNIs and infections [2]. While there is limited direct evidence of concomitant varicella‐zoster virus (VZV) infection and PRES in renal transplant recipients, both conditions share some risk factors. Here, we report a case of PRES and disseminated VZV infection in kidney transplant recipients.

## 2 Case Presentation

A 52‐year‐old woman with end‐stage kidney disease caused by preeclampsia underwent cadaveric renal transplantation after 27 years of hemodialysis. The graft functioned immediately, and immunosuppression therapy with tacrolimus extended‐release (FK‐ER), mycophenolate mofetil (MMF), everolimus, and prednisolone was initiated. Prednisolone was tapered off 3 weeks post‐transplant, and everolimus was also discontinued 1 month post‐transplant due to wound dehiscence. She was maintained on 4.5 mg of FK‐ER (trough level of 7.5 ng/mL), 1000 mg of MMF, with a serum creatinine level of 1.14 mg/dL and blood pressure of 123/75 mmHg.

**FIGURE 1:**
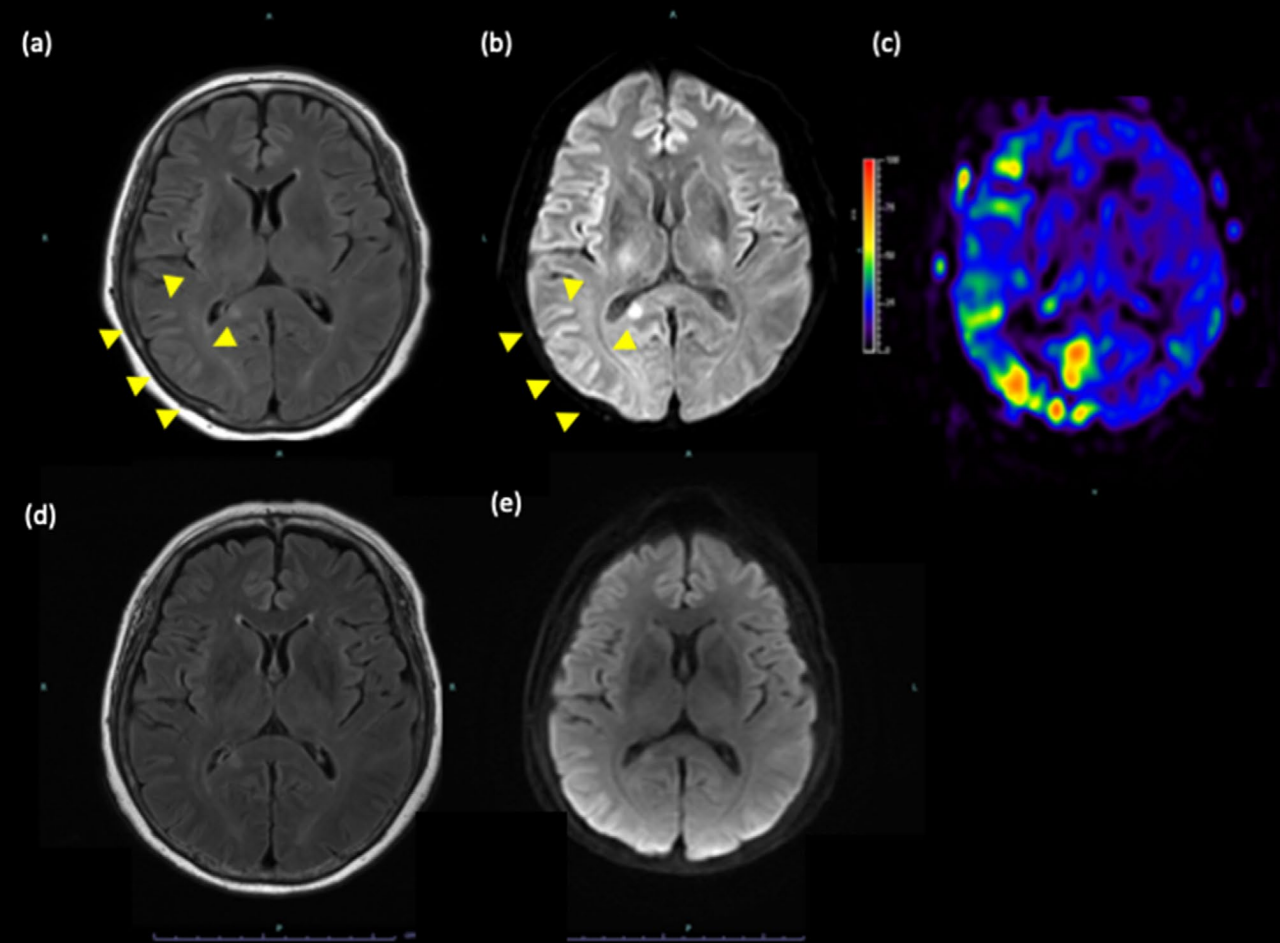
Magnetic resonance images. (a) T2‐Fluid Attenuated Inversion Recovery (FALIR); (b) diffusion‐weighted imaging (DWI); (c) arterial spin labeling (ASL). Initial magnetic resonance imaging revealed hyperintense vasogenic edema in the right occipital and parietal lobes (yellow arrows). Follow‐up magnetic resonance imaging revealed improvement of vasogenic edema on Day 17. (d) FALIR; (e) DWI.

Two months post‐transplant, she presented to the emergency room with headaches, visual disturbances, left upper limb paralysis, and altered consciousness. Her blood pressure was elevated to 176/113 mmHg. Cranial MRI revealed T2 hyperintensities in the right occipital and parietal lobes (Figure 1a,b) consistent with PRES. Arterial spin labeling showed increased blood flow in the right middle and posterior cerebral arteries (Figure 1c). No apparent cerebral hemorrhage or vascular infarction was observed. Cerebrospinal fluid (CSF) examination showed mild increases in protein and glucose without an elevation in cell count. PRES was diagnosed, antihypertensive and anticonvulsant medications were started, and CNI was switched to 140 mg of cyclosporine.

The patient's conscious state improved the following day; however, her headache progressively worsened from Day 3. On Day 5, shingles developed on her right shoulder and flank abdomen. She was diagnosed with disseminated varicella‐zoster virus (VZV) infection and treated with acyclovir at 750 mg/day for 7 days. MMF was reduced to 500 mg. A repeat CSF analysis on Day 12 revealed elevated cell count and protein levels. Despite the initiation of acyclovir, CSF findings were consistent with central nervous system infiltration, necessitating an extension of acyclovir treatment for an additional 7 days, for a total of 14 days. A follow‐up MRI on Day 17 demonstrated the resolution of vasogenic edema (Figure 1d,e). After 30 days, she was discharged without any sequelae. Eight months after disease onset, everolimus was reintroduced. At 30 months post‐transplant, renal function is stable at a creatinine level of 0.86 mg/dL while maintained on cyclosporine (90 mg), MMF (500 mg), and everolimus (1 mg).

## 3 Discussion

PRES is a rare neurological condition that can develop in kidney transplant recipients, primarily due to CNI toxicity and hypertension. CNIs such as tacrolimus and cyclosporine are essential immunosuppressants in transplant management, but their neurotoxicity can lead to PRES. The pathophysiology involves endothelial dysfunction and disruption of the blood–brain barrier, resulting in vasogenic edema [3, 4].

Imaging studies play a vital role in diagnosing PRES. MRI is the preferred modality, as it can detect characteristic findings such as hyperintense lesions on T2‐weighted and fluid‐attenuated inversion recovery (FLAIR) sequences. These lesions are typically located in the posterior regions of the brain but can also involve other areas. In our case, MRI findings were consistent with PRES, showing hyperintense lesions in the occipital and parietal lobes.

Infections can also trigger PRES. While bacterial infections are more commonly associated with PRES, viral infections, including VZV, have been reported in some cases [5, 6, 7]. VZV can cause central nervous system complications, such as vasculopathy and meningitis, which may contribute to the development of PRES. It is believed to be caused by toxin‐mediated endothelial cell dysfunction, resulting in increased vascular permeability and, ultimately, angiogenic edema [8]. In our case, the patient developed a disseminated VZV infection shortly after the onset of PRES; it is possible that this infection may have contributed to the PRES.

We reported 27 Japanese cases of PRES after kidney transplantation (Table 1) [9, 10]. To our knowledge, no cases of concomitant PRES or infection were reported in Japan. The onset ranged from 1 day to 3 years post‐transplantation, and only 18.5% (5/27) of cases occurred after the first month. Given the inconsistencies in reports of normal CNI ranges, we set upper limits of 10 ng/mL for tacrolimus and 200 ng/mL for CsA in our analysis. We observed that 62.5% (10/16) of patients diagnosed with CNI‐associated PRES had levels exceeding these limits. In this case, the trough level was always controlled below 10 ng/mL, but as the discussion was limited to the trough level, the detailed blood concentration could not be examined, and it is possible that an abnormal increase in blood concentration after oral administration caused PRES.

**TABLE 1:** Reported cases of PRES after renal transplantation in Japan.

	*n* = 27
Age, year(range)	12 (3–68)
Gender(female/male), *n*	19/8
Onset, day(range)	4 (1–1095)
Primary disease, *n*	
Hypoplastic/dysplastic kidney	5
Chronic glomerulonephritis	4
Alport syndrome	2
ADPKD	1
ARPKD	1
Branchio‐oto‐renal syndrome	1
Cystic dysplasia of the kidney	1
CHARGE association	1
Diabetic nephropathy	1
FSGS	1
Juvenile nephronophthisis	1
Polycystic kidney	1
Post‐renal failure	1
Preeclampsia	1
Radiation nephritis	1
Unknown	3
Rejection, *n* (%)	4 (14.8%)
Symptoms, *n*	
Seizure	17
Disturbance of consciousness	14
Headache	8
Visual disturbance	
Paralysis	6
Eye fixation	2
Difficulty walking	1
Eye roll up	1
Nausea/vomiting	1
Hypertension, *n* (%)	19 (70%)
CNI(Tac/CsA), *n*	15/10
CNI trough concentration	
High (> 10 ng/mL for tacrolimus, or > 200 ng/mL for CsA)	10
Normal (< 10 ng/mL for tacrolimus, or < 200 ng/mL for CsA)	6
Unknown	11
Brain lesion site, *n*	
Occipital lobe	12
Frontal lobe	11
Parietal lobe	7
Cerebellum	3
Temporal lobe	2
Unknown	11
Treatment, *n*	
Conversion of CNIs	16
Reduction or withdrawal of CNIs	12
Unknown	1

Prognosis for PRES is generally favorable with timely intervention. Most patients recover fully within days to weeks, but delayed diagnosis or inadequate treatment can result in permanent neurological deficits or death [11]. Mortality rates for PRES have been reported at around 19% [12, 13], emphasizing the importance of early recognition and management.

## 4 Conclusion

This case emphasizes the need to recognize PRES as a potential complication in kidney transplant recipients. Infection and CNIs are key triggers. Early diagnosis and treatment adjustments, including modifying immunosuppression and initiating antiviral therapy, are crucial for preventing severe outcomes.

## Consent

Written informed consent was obtained from the patient for publication of this case report and the accompanying images.

## Conflicts of Interest

The authors declare no conflicts of interest.

## References

[1] J. Hinchey , C. Chaves , B. Appignani , et al., “A Reversible Posterior Leukoencephalopathy Syndrome,” New England Journal of Medicine 334 (1996): 494–500. 8559202 10.1056/NEJM199602223340803

[2] W. S. Bartynski , Z. R. Zeigler , R. K. Shadduck , and J. Lister , “Pretransplantation Conditioning Influence on the Occurrence of Cyclosporine or FK‐506 Neurotoxicity in Allogeneic Bone Marrow Transplantation,” AJNR. American Journal of Neuroradiology 25 (2004): 261–269. 14970028 PMC7974616

[3] W. S. Bartynski , H. P. Tan , J. F. Boardman , R. Shapiro , and J. W. Marsh , “Posterior Reversible Encephalopathy Syndrome After Solid Organ Transplantation,” AJNR. American Journal of Neuroradiology 29 (2008): 924–930. 18272559 10.3174/ajnr.A0960 PMC8128592

[4] D. Staykov and S. Schwab , “Posterior Reversible Encephalopathy Syndrome,” Journal of Intensive Care Medicine 27 (2012): 11–24. 21257628 10.1177/0885066610393634

[5] R. C. Seet and A. A. Rabinstein , “Clinical Features and Outcomes of Posterior Reversible Encephalopathy Syndrome Following Bevacizumab Treatment,” QJM 105 (2012): 69–75. 21865314 10.1093/qjmed/hcr139

[6] W. S. Bartynski , J. F. Boardman , Z. R. Zeigler , R. K. Shadduck , and J. Lister , “Posterior Reversible Encephalopathy Syndrome in Infection, Sepsis, and Shock,” AJNR. American Journal of Neuroradiology 27 (2006): 2179–2190. 17110690 PMC7977225

[7] H. Dawood , S. Nasir , M. Ahmed , C. O'Brien , and M. Dawood , “Posterior Reversible Encephalopathy Syndrome Secondary to Varicella Encephalitis,” Cureus 13 (2021): e12484. 33564499 10.7759/cureus.12484 PMC7861057

[8] S. Racchiusa , E. Mormina , A. Ax , O. Musumeci , M. Longo , and F. Granata , “Posterior Reversible Encephalopathy Syndrome (PRES) and Infection: A Systematic Review of the Literature,” Neurological Sciences 40 (2019): 915–922. 30604335 10.1007/s10072-018-3651-4

[9] N. Akutsu , C. Iwashita , M. Maruyama , et al., “Two Cases of Calcineurin Inhibitor‐Associated Reversible Posterior Leukoencephalopathy Syndrome in Renal Transplant Recipients,” Transplantation Proceedings 40 (2008): 2416–2418. 18790253 10.1016/j.transproceed.2008.07.104

[10] Y. Nagano , T. Iwai , M. Tomita , et al., “A Case of Posterior Reversible Encephalopathy Syndrome Developing 10 Years After Kidney Transplant,” Experimental and Clinical Transplantation 20 (2022): 630–632. 35791835 10.6002/ect.2022.0093

[11] T. G. Liman , E. Siebert , and M. Endres , “Posterior Reversible Encephalopathy Syndrome,” Current Opinion in Neurology 32 (2019): 25–35. 30531559 10.1097/WCO.0000000000000640

[12] S. Legriel , O. Schraub , E. Azoulay , et al., “Determinants of Recovery From Severe Posterior Reversible Encephalopathy Syndrome,” PLoS One 7 (2012): e44534. 23024751 10.1371/journal.pone.0044534 PMC3443081

[13] L. M. Alhilali , A. R. Reynolds , and S. Fakhran , “A Multi‐Disciplinary Model of Risk Factors for Fatal Outcome in Posterior Reversible Encephalopathy Syndrome,” Journal of the Neurological Sciences 347 (2014): 59–65. 25271189 10.1016/j.jns.2014.09.019

